# Association of neck circumference and high blood pressure in children and adolescents: a case–control study

**DOI:** 10.1186/s12887-015-0444-2

**Published:** 2015-09-17

**Authors:** Renata Kuciene, Virginija Dulskiene, Jurate Medzioniene

**Affiliations:** Institute of Cardiology, Medical Academy, Lithuanian University of Health Sciences, Sukileliu ave. 17, LT-50009 Kaunas, Lithuania

**Keywords:** Prehypertension, Hypertension, Neck circumference, Overweight, Obesity, Abdominal obesity, Children, Adolescents

## Abstract

**Background:**

High blood pressure (BP) is a serious, common and growing global public health problem. The aim of this study was to evaluate the associations between high NC (neck circumference) alone and in combinations with BMI (body mass index), WC (waist circumference), and high BP among Lithuanian children and adolescents aged 12 to 15 years.

**Methods:**

An epidemiological case–control study was performed between May 2012 and November 2013. NC, WC, hip circumference (HC), mid-upper arm circumference (MUAC), body height, weight, and BP were measured. The participants with high BP (≥90th percentile) were screened on two separate occasions. Data on NC, WC, HC, MUAC, BMI, body adiposity index (BAI), waist-to-height ratio (WHtR), waist-to-hip ratio (WHR), and BP were analyzed in 1947 children and adolescents aged 12–15 years. Age- and sex-adjusted odds ratios (aORs) with 95 % confidence intervals (CI) for the associations were estimated using multivariate logistic regression models.

**Results:**

The prevalence rates of prehypertension (BP ≥90th– < 95th percentile) and hypertension (BP ≥95th percentile) was 6.3 and 25.1 %, respectively. The overall prevalence of high NC (if NC was in the ≥90th percentile), overweight/obesity (as measured by BMI), and abdominal overweight/obesity (if WC was in the ≥75th percentile) were 14.3, 15.8, and 13 %, respectively. After adjustment for age and sex, NC in the ≥90th percentile was significantly associated with an increased risk of elevated BP (prehypertension: aOR = 2.99; 95 % CI, 1.88–4.77; hypertension aOR = 4.05; 95 % CI, 3.03–5.41, and prehypertension/hypertension aOR = 3.75; 95 % CI, 2.86–4.91), compared to the participants with NC in the <90th percentile. Overweight/obesity and abdominal overweight/obesity were also significantly associated with an elevated BP. The combinations including both risk factors (high NC with overweight/obesity, and high NC with abdominal overweight/obesity) showed higher aORs than those with either risk factor alone.

**Conclusions:**

High NC alone—but particularly in combinations with overweight/obesity and abdominal overweight/obesity—was associated with an increased risk of high BP.

## Background

Epidemiological studies have reported that the prevalence of high blood pressure (BP) has significantly increased among children and adolescents in recent years [1–3]. Environmental and genetic factors as well as their interactions are known to affect high BP [4]. A systematic review and meta-analysis (based on findings from 30 cohort studies) have found low-to-moderate tracking of BP from childhood to adulthood [5]. It has also been reported that overweight and obesity in childhood are related to an increased BP and cardiovascular morbidity and mortality in adulthood [6]. High BP is an established risk factor for cardiovascular and circulatory diseases (e.g. ischemic heart disease, stroke, or hypertensive heart disease) [7], and is considered to be the leading cause of death worldwide (responsible for 13 % of deaths globally) [8].

A review of recent meta-analytic studies has shown that general obesity measured by BMI (body mass index), and central or abdominal obesity measured by anthropometric indices such as WC (waist circumference), WHtR (waist-to-height ratio), and WHR (waist-to-hip ratio) are associated with a risk of such cardio-metabolic outcomes as hypertension, dyslipidaemia, fasting plasma glucose concentrations, type 2 diabetes mellitus, and all-cause and cardiovascular disease mortality [9]. NC (neck circumference) has been suggested as an index of the upper body fat distribution [10, 11]. Moreover, NC measurement has been shown to be a simple and time-saving screening measure to identify overweight or obesity [10]. High NC is associated with risk factors for cardiovascular diseases in adults [12–16]. However, few epidemiological studies have examined the associations between high NC and high BP in children and adolescents [17–19].

In Lithuania, a high prevalence of increased BP, or hypertension, is a serious public health problem in children (21.4 %) [20], adolescents (35.1 %) [21], and adult populations [22, 23]. The data of Health Statistics of Lithuania informed that the mortality rate from cardiovascular diseases has remained high in the Lithuanian population over the last decade, and is one of the highest in Europe [24]. Therefore, it is essential to carry out BP and anthropometric measurements and to determine other potential risk factors in Lithuanian children and adolescents for an early identification of subjects who can be at an increased risk for the development of cardiovascular diseases and other chronic non-communicable diseases. Moreover, the associations between high NC and prehypertension and/or hypertension have not been studied among Lithuanian children and adolescents before. Scientific evidence supporting the associations between anthropometric indicators of obesity and other modifiable risk factors and an increased risk of prehypertension and hypertension would be useful for the development of cardiovascular disease prevention strategies, with particular attention to the health of children and adolescents.

The aim of this study was to evaluate the associations between high NC alone as well as in combinations with BMI or WC categories, and the risk of high BP among children and adolescents.

## Methods

### Study population

This case–control study included children and adolescents aged 12 to 15 years who at the time of the examination (from May 2012 to November 2013) attended gymnasiums or secondary schools in Jonava and Prienai district municipalities, which are located in Kaunas County, Lithuania [25]. All the invited schools (*n* = 29) accepted the invitation to participate in the research project. Of 2101 subjects who participated and were examined in the study, 93 subjects were excluded from the statistical analyses because they had any of the following diseases: endocrine diseases, diabetes mellitus, kidney diseases, cardiovascular diseases, or congenital heart defects (information was collected from subjects’ medical records (Form No.027-1/a)). In addition, 61 subjects were excluded due to missing data on anthropometric measurements. Thus, data from 1947 participants were approved for statistical analysis.

Both BP and anthropometric measurements were performed at the participants’ schools by the same team of trained study personnel (physicians and research assistants). A written informed consent was obtained from each participant’s parent or guardian. The study was approved by Kaunas Regional Ethics Committee for Biomedical Research at the Lithuanian University of Health Sciences (protocol No. BE–2–69).

### Measurements

#### Blood pressure measurements

Blood pressure was measured by the physician who was not wearing a white coat in the morning hours (8:30 to 11:30 am). The subjects were advised to avoid tea, coffee, energy drinks, and physical exercises in the morning of the examination day until the measurements were taken. Before the BP measurement, the participants were asked to sit still for 10 min. BP was measured three times with a 5-min rest interval between the measurements, with the subject being in a sitting position; BP was measured using an automatic BP monitor (OMRON M6; OMRON HEALTHCARE CO., LTD, Kyoto, Japan). The average of three BP measurements was calculated. All participants with high BP (BP was in the ≥90th percentile; *n* = 766) during the first screening underwent a second evaluation of BP measurements within a period of 2–3 weeks. If BP was ≥90th percentile during both visits, the final BP status was based on the highest average BP values observed during the first or the second screenings.

Classifications and definitions of BP levels were defined according to “The Fourth Report on the Diagnosis, Evaluation, and Treatment of High Blood Pressure in Children and Adolescents” (National High Blood Pressure Education Program (NHBPEP) Working Group on High Blood Pressure in Children and Adolescents) [26]. According to BP charts for age, sex, and height, normal BP was defined as systolic blood pressure (SBP) and diastolic blood pressure (DBP) below the 90th percentile; prehypertension was defined as an average SBP or DBP levels between ≥90th percentile and <95th percentile; and hypertension was defined as an average SBP or DBP ≥95th percentile.

#### Anthropometric measurements

NC was measured at the level of the thyroid cartilage, with the subject in the standing position and the head held erect. WC was measured at a level midway between the lower rib margin and the iliac crest. Hip circumference (HC) was measured at the maximum circumference around the buttocks. Mid upper arm circumference (MUAC) was measured at a point half way between the elbow and the shoulder. NC, WC, HC, and MUAC were measured with the accuracy of ±0.5 cm using a flexible measuring tape (SECA). Height and weight of the participants (wearing only light clothing and barefooted) were measured with the accuracy of ±0.1 cm and ±0.1 kg, respectively, by using a portable stadiometer and a balance beam scale (SECA measuring equipment).

Cut-off values of NC corresponding to the 90th percentile for the study population were calculated according to the subjects’ age and sex. Values of NC at ≥90th percentile were used to identify subjects with high NC (in boys: ≥35 cm for 12 year-olds, ≥36 cm for 13 year-olds, and ≥38 cm for 14–15 year-olds; in girls: ≥33 cm for 12 year-olds, ≥34 cm for 13–14 year-olds, and ≥35 cm for 15 year-olds). The participants with NC at the <90th percentile were considered to have a normal NC.

BMI was calculated as weight in kilograms divided by the square of height in meters. According to cut-off points of BMI proposed by the International Obesity Task Force [27], the participants were grouped into the following categories of BMI: normal weight, overweight, and obese.

Using the cut-off values of the percentiles of the WC as proposed by the criteria of the Third National Health and Nutrition Examination Survey (NHANES III) [28], the participants were divided into the categories on the basis of their WC: below the 75th percentile (normal waist value), 75th– < 90th percentile (moderate), and ≥90th percentile (high waist value). Using the above-mentioned cut-off values for WC among children and adolescents [28], abdominal obesity was defined as WC ≥90th percentile [28], while we defined abdominal overweight as WC in the 75th– < 90th percentile. Abdominal overweight/obesity among children and adolescents was defined as WC ≥75th percentile. Waist-to-height ratio (WHtR) was calculated as WC (cm) divided by height (cm). Waist-to-hip ratio (WHR) was calculated as WC (cm) divided by HC (cm). Body adiposity index (BAI) was calculated by the following equation reported by Bergman et al. [29]: BAI = (HC (cm)/(height (m)) ^1.5^)–18.

### Statistical analysis

Descriptive statistics (mean and standard deviation (SD)) were computed for the quantitative variables (age, weight, height, BMI, BAI, WC, HC, NC, MUAC, WHtR, WHR, SBP, and DBP). Comparisons between groups were performed by the chi-square (*χ*^2^) test (for categorical variables), *t*-test and ANOVA (for normally distributed continuous variables), and Mann–Whitney *U* test and Kruskal-Wallis test (for non-normally distributed continuous variables). The normality of the distribution of continuous variables was tested by the Kolmogorov-Smirnov test. Pearson’s correlation coefficients were used to examine the associations between anthropometric measurements (NC, BMI, and WC) and SBP and DBP, as well as the associations between NC and BMI, and WC. Univariate and multivariate logistic regression analyses were conducted for both sexes combined to evaluate the associations between NC in the ≥90th percentile, overweight/obesity, abdominal overweight/obesity, and the combinations of NC percentile categories with different status of BMI or WC and the risk of prehypertension, hypertension and prehypertension/hypertension. Crude odds ratios (OR) and adjusted odds ratios (aOR) along with 95 % confidence intervals (CI) were calculated. In multivariate analysis, ORs were adjusted for age and sex.

Statistical analyses were performed using the statistical software package SPSS version 20 for Windows. *P* values <0.05 were considered statistically significant.

## Results

Table 1 presents the characteristics of the study population. Among 1947 study participants aged 12–15 years, 49.4 % (*n* = 962) were boys, and 50.6 % (*n* = 985) were girls. The mean age of all subjects was 13.38 ± 1.09 years (no significant difference in mean age was observed between boys and girls (*P* = 0.850)). Boys were significantly taller, heavier, and had significantly higher mean values of NC, WC, MUAC, WHtR, WHR, and SBP. They had significantly lower mean values of DBP, BAI, and HC than girls did. There was no significant difference in mean BMI between the compared groups.

**Table 1:** Demographic, anthropometric, and BP characteristics of the study participants by sex

Variables	Total (*n* = 1947)	Boys (*n* = 962)	Girls (*n* = 985)	P*
Age (years)	13.38 ± 1.09	13.38 ± 1.11	13.39 ± 1.07	0.850
Height (cm)	163.05 ± 9.64	164.39 ± 11.19	161.75 ± 7.61	<0.001
Weight (kg)	52.88 ± 12.39	53.95 ± 13.70	51.84 ± 10.86	0.004
BMI (kg/m^2^)	19.71 ± 3.37	19.73 ± 3.50	19.69 ± 3.24	0.588
BAI	26.15 ± 3.79	25.37 ± 3.95	26.90 ± 3.46	<0.001
WC (cm)	68.43 ± 8.57	70.29 ± 8.93	66.62 ± 7.79	<0.001
HC (cm)	91.76 ± 8.82	91.20 ± 9.27	92.31 ± 8.32	0.003
NC (cm)	32.21 ± 2.84	33.19 ± 3.06	31.25 ± 2.23	<0.001
MUAC (cm)	26.05 ± 3.26	26.55 ± 3.41	25.56 ± 3.02	<0.001
WHtR	0.42 ± 0.05	0.43 ± 0.05	0.41 ± 0.05	<0.001
WHR	0.75 ± 0.07	0.77 ± 0.06	0.72 ± 0.06	<0.001
SBP (mmHg)	118.45 ± 14.06	121.65 ± 15.65	115.33 ± 11.50	<0.001
DBP (mmHg)	64.21 ± 7.57	63.58 ± 7.71	64.84 ± 7.39	0.001

*BP* blood pressure; *BMI* body mass index; *BAI* body adiposity index; *WC* waist circumference; *HC* hip circumference; *NC* neck circumference; *MUAC* mid-upper arm circumference; *WHtR* waist-to-height ratio; *WHR* waist-to-hip ratio; *SBP* systolic blood pressure; *DBP* diastolic blood pressure

Values are presented as mean ± SD

*Boys versus girls

The general characteristics of the study population according to BP levels are shown in Table 2. The overall prevalence of prehypertension and hypertension was 6.3 and 25.1 % (6.1 and 33.0 % among boys; 6.5 and 17.5 % among girls), respectively. The prevalence of hypertension was higher in boys, while prehypertension rates were higher in girls. In the oldest age group (14–15 years), a greater proportion of the subjects had high BP, compared to the participants who were younger (12–13 years) (38.2 % versus 25.8 %). Overall, 14.3 % of the participants (12.6 % of boys and 15.9 % of girls) had NC equal to or above the 90th percentile. Prehypertension and hypertension were identified in 10.1 and 45.3 % of the participants (9.9 and 61.2 % of boys; 10.2 and 33.1 % of girls) with NC equal to or above the 90th percentile, respectively. The prevalence of overweight, obesity, and overweight/obesity was 12.6, 3.2, and 15.8 % (for boys: 12.7, 4.4, and 17.1 %; for girls: 12.5, 2.0, and 14.5 %), respectively. Among 307 overweight/obese participants, 10.7 % had prehypertension, and 47.2 % had hypertension. The percentage of WC equal to or above the 75th percentile in the entire group of the study subjects was 13.0 % (15.3 % of boys and 10.9 % of girls). Among the participants with abdominal overweight/obesity, there were 10.6 and 47.6 % subjects with prehypertension and hypertension, respectively. The prevalence rates of NC equal to or above the 90th percentile were, accordingly, 7.4 and 50.8 % in the normal weight and the overweight/obesity categories, while they were 8.7 and 51.8 % among subjects with, respectively, WC below the 75th percentile and WC equal to or above the 75th percentile. Obesity-related anthropometric parameters (high NC, overweight/obesity, abdominal overweight/obesity, and the combinations of NC with BMI and NC with WC, including at least one or both of the above-mentioned risk factors) were more prevalent among prehypertensive and hypertensive than among normotensive subjects (Table 2).

**Table 2:** Characteristics of the study participants according to blood pressure levels

Variables	Normotensive (*n* = 1335)		Prehypertensive (*n* = 123)		Hypertensive (*n* = 489)		P*
	n	%	n	%	n	%	
Sex:							
Boys	586	43.9	59	48.0	317	64.8	<0.001
Girls	749	56.1	64	52.0	172	35.2	
Age (years):							
12–13	782	58.6	65	52.8	206	42.1	<0.001
14–15	553	41.4	58	47.2	283	57.9	
NC percentile categories:							
<90th	1211	90.7	95	77.2	363	74.2	<0.001
≥90th	124	9.3	28	22.8	126	25.8	
BMI categories:							
Normal weight	1206	90.3	90	73.2	344	70.3	<0.001
Overweight/obesity	129	9.7	33	26.8	145	29.7	
WC percentile categories:							
<75th	1229	92.1	96	78.0	368	75.3	<0.001
≥75th	106	7.9	27	22.0	121	24.7	
NC and BMI categories:							
NC < 90th and normal weight	1131	84.7	81	65.9	306	62.5	<0.001
NC ≥ 90th and normal weight	75	5.6	9	7.3	38	7.8	
NC < 90th and overweight/obesity	80	6.0	14	11.4	57	11.7	
NC ≥ 90th and overweight/obesity	49	3.7	19	15.4	88	18.0	
NC and WC percentile categories:							
NC < 90th and WC < 75th	1147	85.9	81	65.9	318	65.0	<0.001
NC ≥ 90th and WC < 75th	82	6.2	15	12.1	50	10.2	
NC < 90th and WC ≥75th	64	4.8	14	11.4	45	9.2	
NC ≥ 90th and WC ≥75th	42	3.1	13	10.6	76	15.5	
Age (years)	13.27 ± 1.07		13.41 ± 1.16		13.69 ± 1.06^a, b^		<0.001
Weight (kg)	49.68 ± 10.48		56.23 ± 13.06^a^		60.77 ± 13.25^a, b^		<0.001
Height (cm)	161.47 ± 9.26		163.65 ± 11.02		167.23 ± 9.01^a, b^		<0.001
BMI (kg/m^2^)	18.91 ± 2.81		20.82 ± 3.51^a^		21.62 ± 3.85^a, b^		<0.001
BAI	25.85 ± 3.42		27.07 ± 4.37^a^		26.72 ± 4.44^a, b^		<0.001
NC (cm)	31.49 ± 2.53		33.22 ± 2.68^a^		33.92 ± 2.90^a, b^		<0.001
WC (cm)	66.41 ± 7.11		71.45 ± 9.59^a^		73.19 ± 9.76^a^		<0.001
HC (cm)	89.84 ± 8.08		94.00 ± 8.59^a^		96.45 ± 8.93^a, b^		<0.001
MUAC (cm)	25.28 ± 2.87		27.07 ± 3.60^a^		27.90 ± 3.35^a, b^		<0.001
WHtR	0.41 ± 0.04		0.44 ± 0.05^a^		0.44 ± 0.06^a^		<0.001
WHR	0.74 ± 0.06		0.76 ± 0.07^a^		0.76 ± 0.07^a^		<0.001
SBP (mmHg)	110.75 ± 6.96		123.97 ± 4.29^a^		138.10 ± 9.73^a, b^		<0.001
DBP (mmHg)	62.05 ± 6.38		66.65 ± 7.13^a^		69.51 ± 7.89^a, b^		<0.001

*NC* neck circumference; *BMI* body mass index; *WC* waist circumference; *BAI* body adiposity index; *HC* hip circumferencel; *MUAC* mid-upper arm circumference; *WHtR* waist-to-height ratio; *WHR* waist-to-hip ratio; *SBP* systolic blood pressure; *DBP* diastolic blood pressure

Values are percentages and mean ± SD (standard deviation)

*Significant differences between the groups were determined by the chi-square (*χ* ^2^) test for categorical variables and ANOVA for continuous variables

^a^Significantly different (*P* < 0.05) from normotensive participants

^b^Significantly different (*P* < 0.05) from prehypertensive participants

Prehypertensive and hypertensive subjects (girls and both sexes combined) demonstrated significantly higher mean values of weight, BMI, BAI, WC, HC, NC, MUAC, WHtR, WHR, SBP, and DBP, compared to normotensive participants (Table 2), but there were no significant differences in mean values of BAI and WHR between these groups for boys (data not shown). In boys, the mean values of age, weight, height, BMI, HC, SBP, and DBP were significantly higher in the hypertensive group than in the prehypertensive group, but in girls, no significant difference between these groups in the mean age or any anthropometric parameters was found (data not shown).

The mean values of anthropometric variables including weight, height, BMI, BAI, WC, HC, NC, MUAC, WHtR, WHR, and the mean values of BP (SBP and DBP) increased with increasing NC, BMI, and WC. The highest mean values of SBP and DBP were determined in participants who had both risk factors combined: high NC with overweight/obesity, and high NC with abdominal overweight/obesity (data not shown).

Pearson’s correlation coefficients between NC, BMI, and WC and BP (SBP and DBP) are shown in Table 3. NC, BMI, and WC positively and significantly correlated with BP in boys and in girls, but the correlations of NC and WC with SBP and DBP, and the correlation of BMI with SBP in boys were higher than in girls, while the correlation coefficient of BMI with DBP was higher in girls. NC correlated significantly with BMI (for boys: *r* = 0.593; for girls: *r* = 0.591; for all participants: *r* = 0.555; all *P* values were <0.001)) and WC (for boys: *r* = 0.616; for girls: *r* = 0.606; for all participants: *r* = 0.633; all *P* values were <0.001).

**Table 3:** Pearson’s correlation coefficients between anthropometric parameters and systolic blood pressure and diastolic blood pressure

Variables	NC (cm)	BMI (kg/m^2^)	WC (cm)
*Boys*			
SBP (mmHg)	0.548^a^	0.481^a^	0.446^a^
DBP (mmHg)	0.166^a^	0.181^a^	0.175^a^
*Girls*			
SBP (mmHg)	0.360^a^	0.403^a^	0.372^a^
DBP (mmHg)	0.145^a^	0.226^a^	0.168^a^
*Total*			
SBP (mmHg)	0.518^a^	0.436^a^	0.444^a^
DBP (mmHg)	0.117^a^	0.201^a^	0.149^a^

*NC* neck circumference; *BMI* body mass index; *WC* waist circumference; *SBP* systolic blood pressure; *DBP* diastolic blood pressure

^a^Correlation is significant at the level of 0.01 (2-tailed)

The crude ORs and aORs with 95 % CI for the associations between the selected risk factors and high BP are shown in Table 4.

**Table 4:** Associations between the categories of anthropometric parameters and the risk of high BP (univariate and multivariate analyses)

Variables	Prehypertension		Hypertension		Prehypertension/Hypertension	
	OR (95 % CI)	aOR (95 % CI)	OR (95 % CI)	aOR (95 % CI)	OR (95 % CI)	aOR (95 % CI)
NC percentile categories:						
<90th	1.00	1.00	1.00	1.00	1.00	1.00
≥90th	2.88	2.99	3.39	4.05	3.28	3.75
	(1.82–4.56)	(1.88–4.77)	(2.58–4.46)	(3.03–5.41)	(2.53–4.26)	(2.86–4.91)
BMI categories:						
Normal weight	1.00	1.00	1.00	1.00	1.00	1.00
Overweight/ obesity	3.43	3.53	3.94	4.40	3.83	4.24
	(2.21–5.31)	(2.27–5.48)	(3.02–5.14)	(3.32–5.83)	(2.98–4.93)	(3.26–5.51)
WC percentile categories:						
<75th	1.00	1.00	1.00	1.00	1.00	1.00
≥75th	3.26	3.37	3.81	4.22	3.70	3.97
	(2.04–5.22)	(2.09–5.42)	(2.87–5.07)	(3.12–5.70)	(2.82–4.85)	(2.99–5.26)
NC and BMI categories:						
NC < 90th and normal weight	1.00	1.00	1.00	1.00	1.00	1.00
NC ≥ 90th and normal weight	1.68 ^NS^	1.75^NS^	1.87*	2.26	1.83*	2.12
	(0.81–3.47)	(0.84–3.65)	(1.24–2.82)	(1.48–3.47)	(1.25–2.69)	(1.43–3.15)
NC < 90th and overweight/obesity	2.44*	2.53*	2.63	2.83	2.59	2.83
	(1.33–4.50)	(1.36–4.68)	(1.83–3.78)	(1.93–4.13)	(1.85–3.64)	(1.98–4.02)
NC ≥ 90th and overweight/obesity	5.41	5.52	6.64	8.04	6.38	7.38
	(3.05–9.63)	(3.09–9.84)	(4.58–9.62)	(5.43–11.92)	(4.47–9.12)	(5.09–10.70)
NC and WC percentile categories:						
NC < 90th and WC < 75th	1.00	1.00	1.00	1.00	1.00	1.00
NC ≥ 90th and WC < 75th	2.59*	2.66*	2.20	2.52	2.28	2.56
	(1.43–4.70)	(1.46–4.86)	(1.52–3.19)	(1.70–3.72)	(1.61–3.22)	(1.79–3.66)
NC < 90th and WC ≥ 75th	3.10	3.16	2.54	2.52	2.65	2.68
	(1.67–5.76)	(1.68–5.91)	(1.70–3.79)	(1.65–3.84)	(1.83–3.84)	(1.82–3.94)
NC ≥ 90th and WC ≥ 75th	4.38	4.54	6.53	8.17	6.09	7.06
	(2.26–8.49)	(2.34–8.83)	(4.39–9.71)	(5.37–12.43)	(4.15–8.95)	(4.74–10.51)

*NC* neck circumference; *BMI* body mass index; *WC* waist circumference

*OR* odds ratio; *aOR* adjusted odds ratio for age and sex; *CI* confidence interval

All results were significant at *P* < 0.001, except when noted (*NS* not significant; * – *P* < 0.05)

According to the multivariate models, after adjustment for age and sex, the subjects with high NC had a significant increase in the risk for prehypertension, hypertension, and prehypertension/hypertension (aOR = 2.99, aOR = 4.05, and aOR = 3.75, respectively). Statistically significant associations were found between overweight/obesity and high BP: prehypertension (aOR = 3.53), hypertension (aOR = 4.40), and prehypertension/hypertension (aOR = 4.24). The participants with WC ≥75th percentile had a significantly higher risk of having elevated BP (prehypertension: aOR = 3.37; hypertension: aOR = 4.22; and prehypertension/hypertension aOR = 3.97), if compared to the subjects with WC below the 75th percentile.

Further analyses regarding the associations of the combinations of the categories of anthropometric parameters (NC with BMI; and NC with WC) in relation to the risk of high BP were performed. The subjects in whom these combinations included either or both of the risk factors (high NC or overweight/obesity) had significantly higher aORs for prehypertension, hypertension, and prehypertension/hypertension, except for the combination of NC equal to or above the 90th percentile and normal weight with prehypertension, if compared with the reference category (normal NC with normal weight). When NC and WC were combined, the participants with each combination of risk factors (high NC with non-abdominal overweight/obesity, normal NC with abdominal overweight/obesity, and high NC with abdominal overweight/obesity) demonstrated a significant increase in the risk for prehypertension, hypertension, and prehypertension/hypertension, compared to the combined group of NC below 90th percentile and WC below the 75th percentile. The combinations of high NC with overweight/obesity, and high NC—with abdominal overweight/obesity were associated with an elevated BP at significantly higher aORs (aOR = 7.38 and aOR = 7.06, respectively) than other combinations of obesity-related anthropometric measures with either of the risk factors alone (high NC, overweight/obesity, or abdominal overweight/obesity) were.

## Discussion

To our knowledge, this is the first report that investigated the associations between high NC or the combinations of NC with BMI or WC and elevated BP among Lithuanian schoolchildren aged 12–15 years. Univariate and multivariate logistic regression analyses of our data showed significant associations between high NC and the risk of elevated BP among children and adolescents. The participants with two risk factors in combinations (high NC with overweight/obesity and high NC with abdominal overweight/obesity) had a higher risk of elevated BP, compared to subjects who had either of the risk factors alone.

The data of the present study showed a high prevalence of an elevated BP among Lithuanian schoolchildren. This finding is consistent with findings from other studies conducted on different sample sizes and different age groups of children and adolescents, which also reported a high prevalence of elevated BP [17, 30, 31].

In the current study, 14.3 % of the participants had NC equal to or above the 90th percentile; this percentage is smaller than that observed in the subjects of a cross-sectional study among US children aged 6 to 18 years, where about 24 % of the subjects had high NC (>90th percentile) [17], or in the subjects of another cross-sectional study among Chinese children aged 5–18 years, where about 18 % of the participants had NC equal to or above the 90th percentile [18].

Our data showed that NC correlated significantly with SBP and DBP in both sexes separately and combined. Another recent study [19] demonstrated that NC was positively associated with cardiovascular disease risk factors such as SBP, insulin, and homeostatic model assessment of insulin resistance, and was negatively associated with the quantitative insulin sensitivity check index, fasting glucose to insulin ratio, and serum levels of high-density lipoprotein cholesterol both in bivariate and multivariate analyses conducted in Greek children of both sexes aged 9–13 years. However, in the study by Androutsos et al. [19], it was only in girls that NC positively and significantly correlated with DBP. Besides, NC showed a stronger correlation with SBP than WC did (except for the girls in the present study), whereas WC more strongly correlated with DBP in boys and in both sexes combined than NC did; these findings are partially consistent with the results of the above-mentioned study [19]. In adults of China, the results from a cross-sectional study [14] showed that NC positively correlated with SBP and DBP, fasting blood glucose levels, and triglyceride concentrations, and negatively correlated with high density lipoprotein cholesterol levels in both sexes separately. A recent study by Stabe et al. [16] has estimated that NC was positively associated with the metabolic syndrome, insulin resistance, and abdominal visceral fat. The findings from the Framingham Heart Study [13] showed that NC was associated with cardiovascular disease risk factors; these results were obtained after adjustment for the levels of visceral adipose tissue. It has been established that visceral adipose tissue was more strongly associated with metabolic risk factors than subcutaneous abdominal adipose tissue was [32]. Elevated levels of free fatty acids cause obesity-related insulin resistance and cardiovascular disease [33].

Previous studies that have investigated the association between high NC and BMI and elevated BP among children and adolescents have reported different findings [17, 18]. A cross-sectional study in the United States reported a significantly higher risk for elevated BP in the participants with high NC (NC above the 90th percentile) than in those with normal NC within each BMI category (normal weight: OR = 1.78; overweight: OR = 2.74; obese: OR = 2.44) [17]. In another cross-sectional study in China, among the subjects with normal BMI, high NC (NC equal to or above the 90th percentile) was significantly associated with an increased risk of prehypertension (aOR = 1.44) after adjustment for age, sex, BMI, and WC, but no significant aORs were found in either overweight or obese categories [18]. Meanwhile, the current study investigated the associations between high NC alone or in combinations with overweight/obesity or abdominal overweight/obesity, and the risk of high BP. We found significant associations between NC equal to or above the 90th percentile and elevated BP in both sexes combined. Besides, our data indicated the highest aORs of prehypertension, hypertension, and prehypertension/hypertension in subjects with both risk factors combined as compared to those with either of the risk factors alone. The prevalence of high NC increased with the increasing BMI category, and this is in agreement with several other studies [17, 18]. The current study also showed that NC positively correlated with WC and BMI, and these findings are concordant with the findings from a previous study conducted among children and adolescents [34].

According to our data, overweight/obese subjects have a significantly higher risk of elevated BP compared to those with normal weight. Another study also found that overweight/obesity was associated with prehypertension and hypertension in children and adolescents aged 6–16 years [35]. Cardiovascular risk factors (high BP, elevated levels of total cholesterol, low-density lipoprotein cholesterol, and triglycerides) are more prevalent among overweight/obese children and adolescents than among subjects with normal weight [36].

The results of the current study showed that WC equal to or above the 75th percentile was significantly associated with an increased risk of high BP. The study by Savva et al. [37] found that children (aged 10–14 years) with WC above the 75th percentile had significantly higher mean values of SBP and DBP, and higher levels of triglycerides, low-density lipoprotein cholesterol, and total cholesterol, compared with those with WC equal to or below the 75th percentile. Guimarães et al. [38] showed that adolescents (aged 11–18 years) with WC above the 75th percentile had a significantly higher risk for high SBP, but not significantly—for high DBP, compared to the participants who had WC equal to or below the 75th percentile. In contrast to our findings, Moser et al. [39] did not observe any significant association between abdominal obesity (WC equal to or above the 75th percentile) and high BP in children and adolescents aged from 10 to 16 years in Brazil.

The data from the study by LaBerge et al. [40] confirmed that NC measurements have very good inter- and intra-rater reliability and, consequently, they do not require multiple repeated measurements for precision and reliability. NC measurement is cheaper and even easier to perform comparing with measurement of WC, which can change during the day [41]. However, there is no consensus regarding the general protocols for the measurements of NC [16] and WC [9], and there are no accurate cut-offs values for children and adolescents to define high NC. Research studies reported that NC as an index of the upper body fat distribution [10] was associated with cardiometabolic risk factors [12, 16]. As BMI is a weight-for-height measure [9], it does not distinguish between fat mass and lean mass [42]. Meanwhile, WC measurements cannot differentiate between visceral adipose tissue and subcutaneous adipose tissue [43]. However, Brambilla et al. [44] analyzed the relationship between anthropometry and visceral and subcutaneous adipose tissue as measured by magnetic resonance imaging in children and adolescents aged 7–16 years, and found that WC may be a good predictor of visceral adipose tissue, and BMI—a predictor of subcutaneous adipose tissue. Scientific studies reported that WC was a better predictor and indicator of cardiovascular disease risk factors in children and adolescents than BMI was [37, 45]. The findings of the current study showed the importance of the interactions of different anthropometric indicators of obesity in assessing the risk of high BP. Indeed, high NC with in combinations with overweight/obesity and abdominal overweight/obesity can more accurately assess cardiovascular risk in children and adolescents than high NC alone. Data of other research studies [45, 46] also demonstrated that combinations including both categories of obesity indicated by different anthropometric measurements (e.g. BMI and WC) are associated with a higher risk of elevated BP compared to either of the risk factors alone.

Our study has several limitations. The current study examined only a sample of 12–15 year-old children and adolescents. Therefore, our findings need to be confirmed and extended in further larger or collaborative studies among children and adolescent populations. In the current study, BP readings were obtained by an automatic oscillometric BP monitor, although, according to the Fourth Report, high BP readings obtained with an oscillometric device should be repeated by using auscultation [26]. While there is no accurate consensus on NC cut-off values that define high NC among children and adolescents, we used the cut-off values of the 90th percentile of NC in our study sample. The comparison of findings of the current study and other published studies is not easy because of differences in sample size, the age of the investigated children and adolescents, the number of BP measurements, the cut-off values for defining high NC, and the potential confounders. Categories of overweight and obesity were placed into a single category (overweight/obesity) due to the small number of the study subjects in the obesity group. Further research is required to analyze the interaction between high NC and high BMI in more BMI subgroups. In the current study, there was no adjustment for family history of hypertension, pubertal status, socioeconomic factors, the intensity of physical activity, nutrition habits, smoking status, or other potential confounding factors because information on these risk factors was lacking. Another limitation of our study is that biochemical parameters, genetic factors, and pubertal status were not assessed. Furthermore, inter-observer coefficient of variation was not investigated in our research. Our future research should analyze the associations between high BP and many different risk factors.

Despite these limitations, the results of the present study showed that the prevalence of elevated BP is high among Lithuanian schoolchildren, and significant associations were found between the selected anthropometric indicators of obesity and the risk of high BP. Consequently, public health strategies in Lithuania should focus more on the prevention and control of the risk factors of cardiovascular diseases. The efforts of persistent behavioral changes related to healthy nutrition, increased physical activity, and reduced unhealthy behaviors for preventing and controlling overweight, obesity, and high BP may decrease the risk of cardiovascular disease.

## Conclusions

The results from this study indicated a high prevalence of elevated BP among 12–15 year-old Lithuanian schoolchildren. After adjusting for age and sex, high NC was significantly associated with the risk of prehypertension and hypertension; moreover, the combinations of high NC with overweight/obesity and high NC with abdominal overweight/obesity may be preferable to high NC alone for risk assessment of high BP. NC measurement could be used in clinical practice and in research settings.

## Abbreviations

AOR: Adjusted odds ratio
BAI: Body adiposity index
BMI: Body mass index
BP: Blood pressure
CI: Confidence interval
DBP: Diastolic blood pressure
HC: Hip circumference
MUAC: Mid-upper arm circumference
NC: Neck circumference
OR: Odds ratio
SBP: Systolic blood pressure
SD: Standard deviation
WC: Waist circumference
WHR: Waist-to-hip ratio
WHtR: Waist-to-height ratio
